# Event- and time-dependent decline of outcome information in the primate prefrontal cortex

**DOI:** 10.1038/srep25622

**Published:** 2016-05-10

**Authors:** Encarni Marcos, Satoshi Tsujimoto, Aldo Genovesio

**Affiliations:** 1 Department of Physiology and Pharmacology, Sapienza University of Rome, Rome, Italy; 2 Department of Intelligence Science and Technology, Graduate School of Informatics, Kyoto University, Kyoto, Japan; 3 Nielsen Consumer Neuroscience, Tokyo, Japan

## Abstract

The prefrontal cortex (PF) is involved in outcome-based flexible adaptation in a dynamically changing environment. The outcome signal dissipates gradually over time, but the temporal dynamics of this dissipation remains unknown. To examine this issue, we analyzed the outcome-related activity of PF neurons in 2 monkeys in a distance discrimination task. The initial prestimulus period of this task varied in duration, allowing us to dissociate the effects of time and event on the decline in previous outcome-related activity —previous correct versus previous error. We observed 2 types of decline in previous outcome representation: PF neurons that ceased to encode the previous outcome as time passed (time-dependent) and neurons that maintained their signal but it decreased rapidly after the occurrence of a new external event (event-dependent). Although the time-dependent dynamics explained the decline in a greater proportion of neurons, the event-dependent decline was also observed in a significant population of neurons.

Most neurophysiological studies on working memory have examined how information, such as items, rules, and motor plans, persist over time in the prefrontal cortex (PF)^1^,^2^. One unknown aspect of the mechanisms of working memory has been how information can be held in memory after it is presented, prompting whether the same neurons participate throughout the duration of a delay in working memory to be studied.

To this end, Brody *et al.* identified 2 categories of neurons in the PF that maintain the frequency of a mechanical vibration for a delay of 3 or 6 seconds: one population with persistent activity and another population with nonpersistent activity that is modulated in the early or late part of the delay, showing a variety of encoding schemes^3^. Even the activity of cells that are involved for the entire delay exhibit variations in the timing of their activity. Single-trial neural responses advance through a sequence of stable states that are evoked by the presence of new task events or intrinsically generated by the neural network^4^,^5^,^6^. More recent neurophysiological studies in nonhuman primates have advanced past the trial’s temporal window, asking whether and in which circumstances information not only persists during a delay but also modulates the neural activity in the following trials^7^,^8^,^9^,^10^. Complementary studies in humans and other animals, beyond primates, have shown the same persistent activity^11^,^12^,^13^. In contrast to the delay period that is used in working memory tasks in which information should be maintained, the neural encoding of previous choices and outcome gradually declines after reward delivery but can still be represented in subsequent trials. We have previously shown that goal and outcome information remain encoded in the subsequent trial, even when they are irrelevant to task performance^9^,^14^. However, the temporal dynamics of the decline of outcome signals remain unknown. In this study, we examine this issue using the dataset that was collected by Genovesio *et al.*^14^,^15^. The outcome signal is one of the most interesting signals to characterize, because it represents the strongest signal that persists from one trial to the next. We addressed the question of whether neurons cease to encode past outcome information merely due to the passage of time—i.e., a gradual temporal dissipation of information—or whether they do so as an effect of new task demands.

We analyzed the activity of neurons in the dorsolateral PF (PFdl) and caudal periarcuate (PA) in 2 rhesus monkeys that performed a distance discrimination task^15^ (Fig. 1). The monkeys were required to report which of 2 visual stimuli—differing in shape and color—sequentially presented, was farther from a central reference point. Thus, while information such as the absolute distance of each stimulus from the reference point or their visual features is critical for a correct performance of any given trial, recent past choices or past outcomes are irrelevant. Yet, neurons in the PF represent the recent previous outcome (previous trial correct vs incorrect) in their activity^14^.

In this study, we aimed to increase our understanding of the dynamics of how outcome information fades in the following trial, capitalizing on a key feature of our task: a variable prestimulus period. A prestimulus duration of 400 ms or 800 ms separated the beginning of a trial from the presentation of the first stimulus (S1). The difference in prestimulus duration provides a suitable framework to examine the dynamics of the representation of outcome information by PFdl neurons, which has been precluded in previous studies that have implemented fixed durations^8^,^9^,^16^,^17^. For example, in the strategy task in Genovesio *et al.*^9^,^18^, a constant fixation duration before the presentation of a stimulus prevented the timing and event factors from being dissociated. Thus, using this newer distance discrimination paradigm allows us to distinguish between the influence of the passage of time per se on the decline of past information and the impact of the appearance of a new event, as represented by the presentation of a stimulus.

## Results

Overall, the 2 monkeys performed the task accurately: 78% for Monkey 1 and 79% for Monkey 2. To analyze the dynamics of the neurons that encoded the previous outcome, we divided the trials into 2 groups, based on the prestimulus duration: 400 ms (*Pre-S 400* trials) and 800 ms (*Pre-S 800* trials). Then, we divided the first 1200 ms of a trial into 3 periods, depending on the type of trial: “early prestimulus”, “early S1”, and “late S1” periods for *Pre-S 400* trials and “early prestimulus”, “late prestimulus”, and “early S1” periods for *Pre-S 800* trials (see Materials and Methods). During the early prestimulus period, 25.6% of neurons (N = 369/1443) were modulated by the previous outcome (correct or incorrect response) in *Pre-S 400* trials versus 25.8% (N = 372/1443) in *Pre-S 800* trials.

To examine the dynamics of the neural outcome signal of these neurons, we identified the neurons that maintained the outcome modulation during the periods of interest in the 2 types of trials. The number of neurons that encoded the previous outcome decreased over time (Fig. 2A). Notably, 800 ms after the prestimulus onset, which corresponds to the start of the trial, the proportion of outcome-dependent neurons was higher in *Pre-S 800* than in *Pre-S 400* trials (16.9% vs 4.9%), possibly related to the disparate times of the onset of S1, after which certain neurons might lose their previous outcome selectivity due to their involvement in new aspects of the task.

To identify the neurons that ceased encoding the previous outcome after the appearance of an external event—represented by the onset of S1—or due the passage of time, we divided the cells into 3 groups: *event-dependent*, *time-dependent 400,* and *time-dependent 800* neurons (see Materials and Methods). To differentiate the 3 subpopulations, we first selected the group of neurons whose activity was modulated by the previous outcome in the early prestimulus period in the *Pre-S 400* and *Pre-S 800* trials—213 neurons were common to both trials, of which 125 (58.69%) had a preference (higher activity) for a previous incorrect outcome, whereas the remaining 88 neurons preferred a previous correct outcome (41.31%). Next, from this subset, we determined the event- or time-dependent neurons. The activity of 31 cells waned after S1 onset and were classified as *event-dependent* neurons (Fig. 2B,C). In this subset, the percentage of neurons that preferred correct and incorrect previous outcome was similar to that in the larger group (38.71% of neurons had a preferred previous correct outcome). Conversely, the previous outcome selectivity faded in 68 neurons 400 ms after the beginning of the trial (44.12% preferred a previous correct trial) independently of the time of S1 onset; 28 neurons showed this effect 800 ms after the beginning of the trial (57.14% preferred a previous correct trial) (Fig. 2B,C). These groups of neurons were considered *time-dependent 400* and *time-dependent 800* neurons, respectively. Thus, from the original group, we could successfully classify 127 neurons (N = 127/213; 59.62%) as either *event-dependent* (24.41%) or *time-dependent* (75.59%) neurons (Fig. 2C). Of the remaining 81 neurons that still coded a previous outcome 800 ms after the start of the trial (18 neurons for Pre-S 400 trials and 63 neurons for Pre-S 800 trials), 9 neurons (~11.1%) still coded it after the disappearance of S1. Thus, most of the neurons ceased to code the previous outcome in the first half of the new trial.

Event- and time-dependent neurons were distributed over the PFdl and PA, with more of the latter group present in both areas (89.5% in PF and 73.1% in PA). Notably, the behavior of the 2 monkeys in sessions with event- or time-dependent neurons did not significantly differ between them in terms of proportion of correct trials or reaction time (RT) (Monkey 1: 75% vs. 79% and 367 ± 6 ms (mean ± SEM) vs. 361 ± 7 ms; Monkey 2: 78% vs. 78% and 402 ± 14 ms vs. 401 ± 5 ms; two sample t-test, p > 0.05). Thus, the event or time-dependent decline observed in the neural responses was not due to a difference in the monkeys’ behavior between sessions.

Figure 3 shows the activity of 2 examples of previous outcome-selective neurons. Figure 3A shows a neuron with higher activity for previous incorrect trial than for previous correct one that was classified as an *event-dependent* neuron, with activity aligned to the S1 onset for *Pre-S 400* and *Pre-S 800* trials. The neuronal activity was significantly modulated by the previous outcome in the early prestimulus period in the *Pre-S 400* trials and in the early and late prestimulus periods in the *Pre-S 800* trials. Its modulation ceased only after the onset of S1, indicating that the cease in the coding of previous outcome was event-dependent. Figure 3B shows an example of a *time-dependent 400* neuron with higher activity for previous correct trial than for previous incorrect one. Its response was modulated by the previous outcome in the early prestimulus period in the *Pre-S 400* and *Pre-S 800* trials but not in the late prestimulus period of *Pre-S 800* trials. Thus, in *Pre-S 400* trials, the neuron ceased to encode the previous outcome after the presentation of S1— and in Pre-S 800 trials it did so 400 ms after the start of a trial. This result indicates that the decline in previous outcome coding does not depend on the onset of S1 but is instead a function of time.

The mean firing rate of the 3 populations of neurons, with trials divided by *Pre-S 400* and *Pre-S 800* trials, is shown in Fig. 4. Figure 4A shows that *event-dependent* neurons had a significantly different response for preferred and nonpreferred previous outcome that remained during the entire prestimulus period for *Pre-S 400* and *Pre-S 800* trials. After the presentation of S1, however, the mean firing rate of these neurons became previous outcome-independent. Figure 4B shows the population activity for the other category of neurons: *time-dependent* neurons. The *time-dependent 400* neurons showed a decline in the previous outcome modulation in the early S1 period during *Pre-S 400* trials and in the late prestimulus period during *Pre-S 800* trials and therefore it occurs independently of the onset of S1. Similarly, the *time-dependent 800* population of neurons lost the previous outcome signal 800 ms after the start of the trial and thus the activity decline was unrelated to S1 onset. We also examined whether there were differences in the selectivity indexes between populations of neurons. Neurons that exhibited an event- or time-dependent decline did not show a significant difference between their selectivity indexes (0.50 ± 0.04 (mean ± SEM) and 0.47 ± 0.02, respectively; two-sample t-test, p = 0.64).

Next, we tested whether neurons differed in their involvement in various aspects of the task after the presentation of S1. To this end, we quantified the neurons from each group (N = 31 and N = 96) that, after the presentation of S1, encoded the visual features (color/shape) of S1 or its absolute distance from the central reference point. We found that 12.9% and 6.5% of *event-dependent* neurons encoded the color of S1 and its distance, respectively. In the *time-dependent* neural population, 11.5% of the neurons encoded the color and 12.5% encoded the distance. Notably, the proportion of neurons that encoded the absolute distance of S1, but not its visual features, was higher in the *time-dependent* neurons than in the *event-dependent* group. This result suggests a potential difference in the function between groups, with *time-dependent* neurons being more involved in the distance-encoding than the *event-dependent* subset.

## Discussion

Our results show that the representation of previous outcome by neurons in the PF wanes not only as an effect of time but also due to the presentation of an external event that is represented by the first stimulus.

Outcome signals have been reported in several cortical areas, including the orbitofrontal^19^,^20^, cingulate cortex^21^, and subcortical areas, such as the striatum^8^. Focusing on the PFdl, several studies have shown evidence of reward-coding in terms of expectancy^22^,^23^,^24^,^25^ and outcome^26^,^27^. In particular, outcome signals have been reported in the PFdl not only in the period immediately after reward delivery^26^,^27^ but also later in the following trial^7^,^14^. In this study, we examined the decline of such outcome-related signals, focusing our analyses on the neural representation of the previous outcome in the first part of a trial. The 2 prestimulus durations used in our experiment allowed us to distinguish neurons with an event-dependent decline from those with a time-dependent decline.

Experimental and theoretical work has provided evidence that cortical neurons coordinate to transiently and sequentially process the information in a task^4^,^28^,^29^,^30^. For example, neurophysiological studies have shown that neurons in the PF transit through various states, depending on the current task-relevant information or demands^31^,^32^. Similarly, our results indicate that the neural representation of outcome information declines gradually not only due to the passage of time but also triggered by new events such as the presentation of a stimulus. This result is consistent with the finding that neurons change states as task-relevant information appears, encoding it in the relevant period and, in certain instances, in subsequent periods^33^,^34^. However, when task periods have constant durations, such as in Takeda and Funahashi^33^, the impact of time or the impact of the appearance of a new external event on the transitions between neural states cannot be disambiguated.

We have previously shown that the previous trial outcome is not only represented by the neural activity during the first part of a trial but it can also appear later^14^. In the current study, we focused solely on the first portion of the trial, because it is in this period in which most of the neurons that encoded the previous outcome were identified. In addition, the 2 prestimulus durations enabled us to make a clear distinction between the influence of time and event on the neural representation of a previous outcome. Thus, the prestimulus and early poststimulus periods represent suitable windows to properly examine the temporal dynamics following the representation of a previous outcome. With this procedure, we could classify nearly 60% of neurons that showed previous outcome selectivity at the beginning of the trial. The remaining neurons could not be identified as having undergone an event- or time-dependent decline, based on our period of interest. These neurons need to be classified in successive periods of the trial in which, unfortunately, the effects of time and event are not entirely separable. In subsequent periods of the trial, additional elements, such as relative time, might play a role in the neural response dynamics of the representation of a previous outcome, the examination of which was beyond the scope of this study.

An open question is why previous outcome information needs to be maintained. To adapt to changing environments, humans and other animals constantly alter their strategy in a way described by a reinforcement learning theory^35^, in which future choices are selected, based on the outcomes that are expected from previous experience. Neurons from the PF that have been recorded while monkeys perform simulated competitive games^36^ modulate their activity in relation to the choice and outcome that are experienced not only in the preceding trial but also in several trials before^7^,^16^. Thus, in cases in which choices and outcomes are critical for the performance of a task, those signals are encoded by neurons, even many trials after they occur. We have previously shown that the PF does not indifferently track all past irrelevant information^14^. While neurons maintained irrelevant information such as previous outcome and goals, they did not code other information such as the previous position or previous color and shape of the second stimulus. Continuous monitoring of goal and outcome, even when irrelevant, might support, among other functions, the exploration of appropriate strategies that could, for example, speed up learning in conditional motor paradigms^18^,^37^. In our task, the information on previous outcome was neither critical nor necessary to correctly perform a trial and thus it provided a great advantage for measuring the decline of its neural representation —i.e., the previous outcome is irrelevant at all times, and thus, the presence of a new event, such as the presentation of S1, does not diminish its importance in the trial.

In summary, our results indicate that signals, such as previous outcome, can decline in the PF as a consequence of time and new events. It is possible that new events might trigger the decline by changing the coalition of neurons that work together^38^. Future studies should determine the generality of our results by examining the encoding and decline of other types of information and identify the underlying factors that elicit the transition between neural states when they are caused by the passage of time or by changes in the task events.

## Materials and Methods

### Procedures

Our procedures followed the Guide for the Care and Use of Laboratory Animals (1996, ISBN 0-309-05377-3) and were approved by the NIMH Animal Care and Use Committee.

### Behavioral task

**Figure 1:**
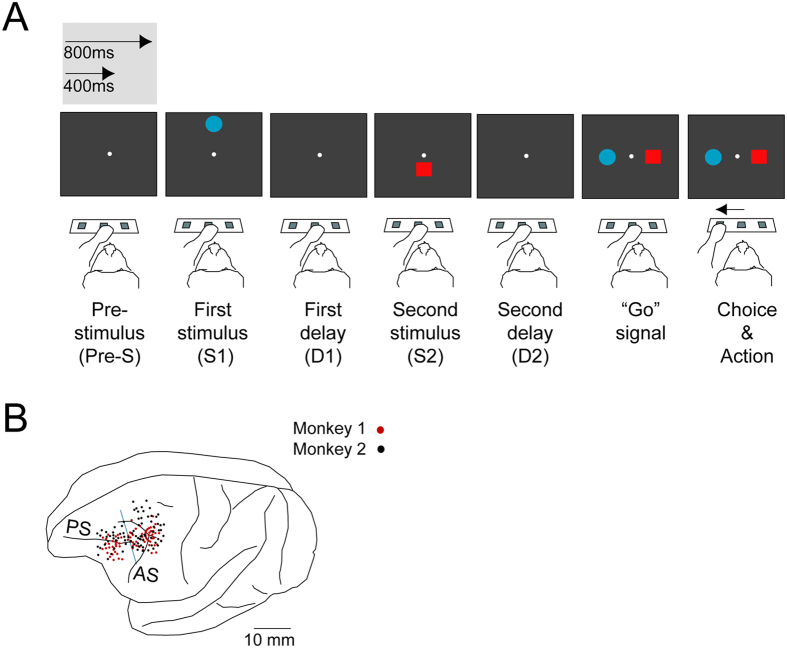
Experimental task and recording locations. (**A**) Order of events during a trial. Each trial starts with the presentation of a central reference point. A prestimulus period of 400 ms or 800 ms separates the start of a trial from the presentation of a first stimulus (S1). S1 is presented for 1.0 s and is followed by a first delay (D1). Subsequently, a second stimulus (S2) appears for 1.0 s. A second delay (D2) separates the presentation of S2 from the reappearance of the 2 stimuli (goals), which instructs the monkeys to select one of them (“go” signal). To get the reward, the monkeys must select the goal that was farther from the central reference point. Our analyses are performed with the trials sorted by the duration of the prestimulus period. (**B**) Composite illustration of penetration sites in both monkeys, relative to sulcal landmarks. The vertical blue line indicates the division between the dorsolateral prefrontal (left) and periarcuate (right) areas. AS, arcuate sulcus and PS, principal sulcus.

**Figure 2:**
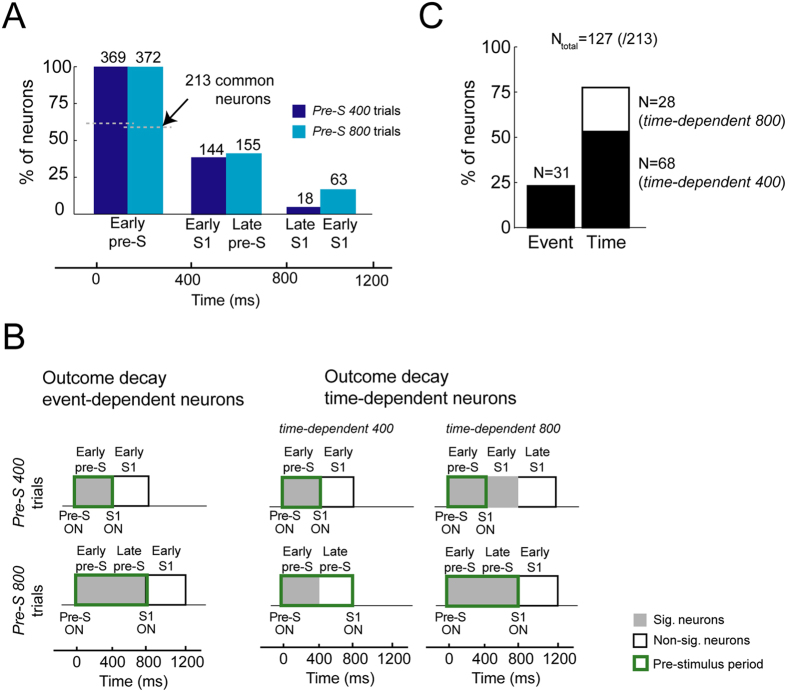
Previous outcome-selective neurons. (**A**) Percentage of previous outcome-selective neurons calculated in the 3 trial periods for each trial type, 400 ms apart. Trials are divided by the duration of the prestimulus period (400 ms or 800 ms, referred to as *Pre-S 400* and *Pre-S 800* trials, respectively). The analyses in the remaining periods were performed considering the initial group of neurons identified for each trial type (N = 369 for *Pre-S 400* trials and N = 372 for *Pre-S 800* trials). From the total number of neurons selective for the previous outcome in the early prestimulus (pre-S) period in the *Pre-S 400* and *Pre-S 800* trials, we identified 213 neurons that were common to both groups. (**B**) Classification of the outcome decline *event-dependent* and outcome decline *time-dependent* neurons. Neurons were classified as *event-dependent* if they showed a significant previous outcome modulation during the entire prestimulus period (gray rectangle) but not after the onset of S1 (white rectangle). Neurons were classified as *time-dependent* neurons if they ceased to represent the previous outcome due to the passage of time. Neurons were classified as *time-dependent 400* neurons if they ceased to encode the previous outcome 400 ms after the start of the trial or *time-dependent 800* neurons if they did so 800 ms after the start. In both cases, the dynamics of the neural representation of the previous outcome signal was not dependent on the presentation of S1. (**C**) Percentage of the neurons classified in each group. A total of 127 neurons (of 213) could be classified as *event-dependent* (24.41%) or *time-dependent* (53.54% *time-dependent 400* and 22.05% *time-dependent 800*) neurons.

**Figure 3:**
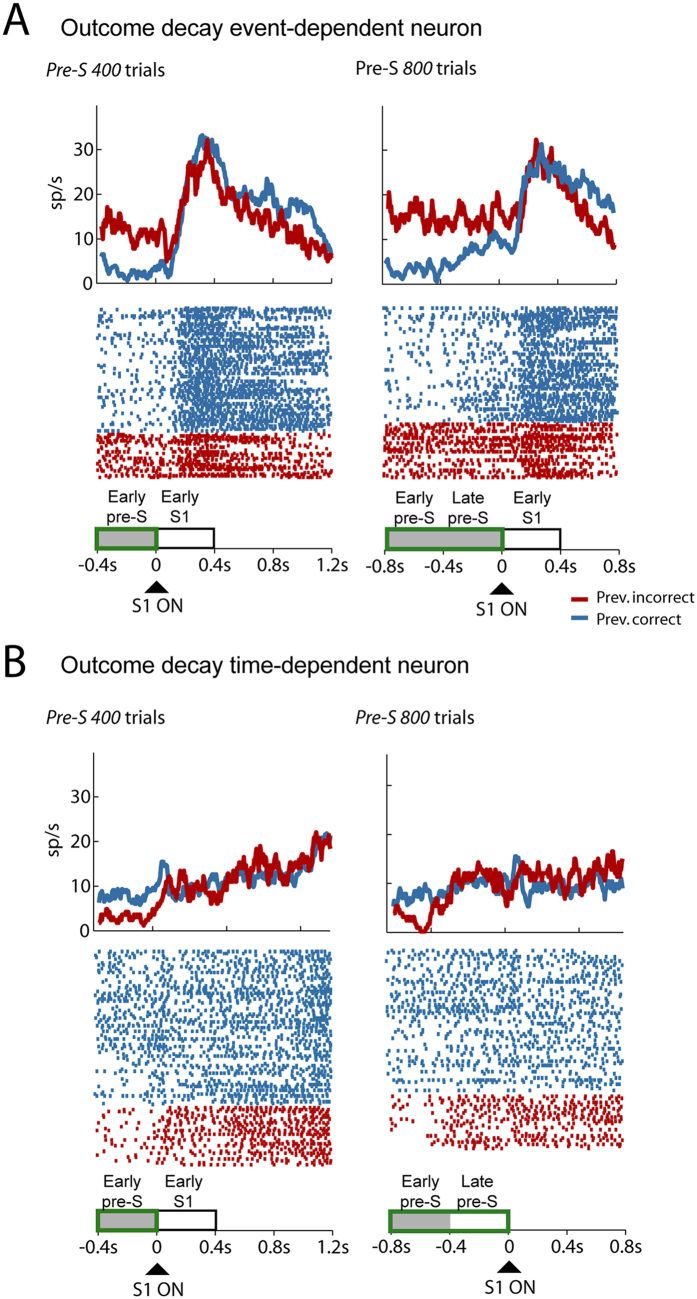
Raster plot of two example neurons with different previous outcome decline dynamics. (**A**) Mean firing rate activity (*Top panel*) and spike times (*Bottom panel*) of a neuron showing an *event-dependent* decline of previous outcome-encoding. The neuron showed higher activity frequency when the previous trial was an incorrect trial (red) than when it was a correct one (blue). After presentation of S1, the neuron did not show a previous outcome modulation for *Pre-S 400* (*Left panel*) or *Pre-S 800* (*Right panel*) trials. (**B**) Mean firing rate activity (*Top panel*) and spike times (*Bottom panel*) of a neuron showing a *time-dependent* decline of previous outcome-encoding. The neuron showed higher activity for previous correct (blue) than for previous incorrect trials (red). The neuron stopped encoding the previous outcome 400 ms after the start of the trial in *Pre-S 400* (*Left panel*) and *Pre-S 800* (*Right panel*) trials.

**Figure 4:**
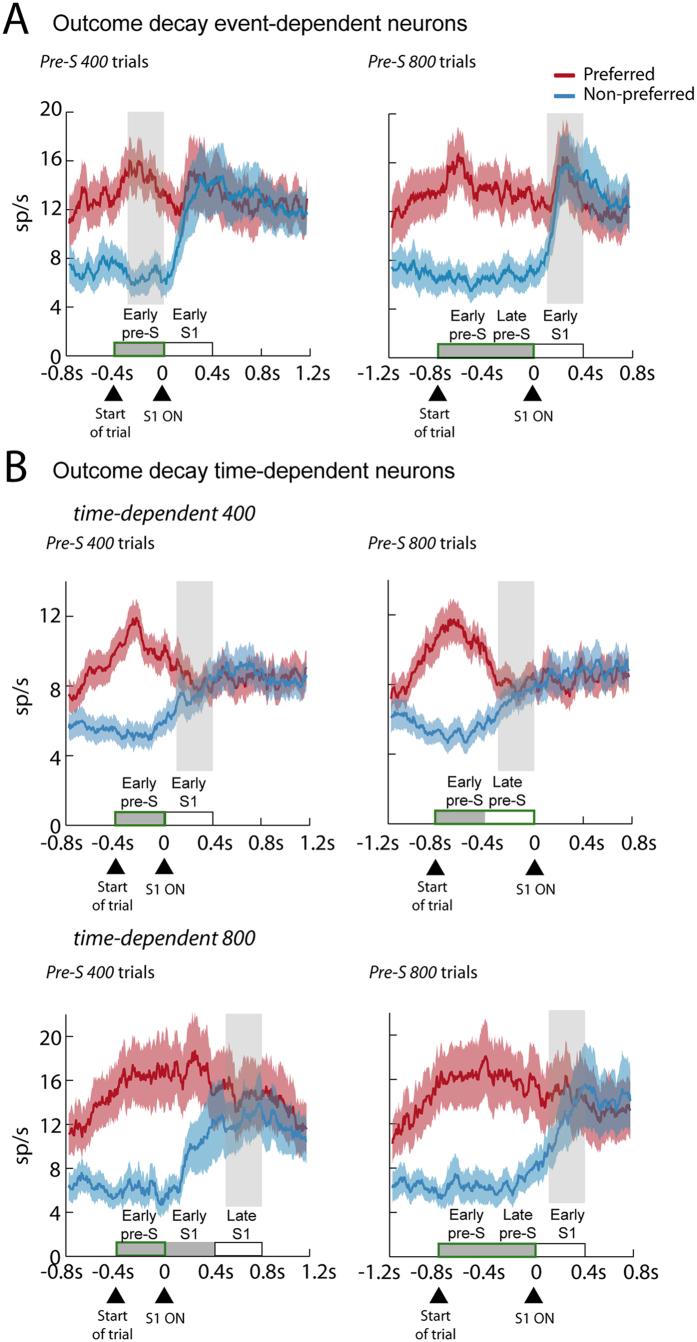
Mean firing rate of different classes of neurons aligned to presentation of S1 for *Pre-S 400* (*Left panels*) and *Pre-S 800* (*Right panels*) trials. (**A**) Mean activity of *event-dependent* neurons (N = 31). The neurons had a significant previous outcome modulation, represented by the difference between preferred and nonpreferred previous outcome before and during the prestimulus period. The encoding was independent of the prestimulus duration and disappearead after the presentation of S1. (**B**) Mean activity of *time-dependent* neurons. *Top panel*, mean response of *time-dependent 400* neurons (N = 68). These neurons showed a previous outcome modulation before and during the early prestimulus period but not later. *Bottom panel*, mean response of *time-dependent 800* neurons (N = 28). These neurons encoded the previous outcome before and during the early prestimulus period of both types of trials, in the late prestimulus of the *Pre-S 800* trials, and in the early S1 period of the *Pre-S 400* trials. In summary, *time-dependent 800* neurons were outcome-modulated in the first 800 ms after the beginning of the trial, independently of the onset of S1. Shaded areas are SEM in all cases.

Two male rhesus monkeys (*Macaca Mulatta*; 8 and 8.5 kg) performed the distance discrimination task (Fig. 1A) while neurons from their PFdl and PA were recorded (Fig. 1B). Details on the experimental task have been reported by Genovesio *et al.*^15^. In short, the monkeys were required to select which of 2 visual stimuli that were presented sequentially was farther from a reference point at the center of a screen. A trial began when the monkey pressed with its left hand the center switch (key) of a series of 3 × 2-cm infrared switches that were located within reach under the video screen, leading to the appearance of the reference point. After a prestimulus period of 400 or 800 ms, the first visual stimulus (S1) appeared above or below (up/down) the reference point for 1.0 s. A delay (D1) of 400 or 800 ms separated the presentation of S1 from the appearance of the second stimulus (S2), which also lasted 1.0 s. S2 appeared above the reference point if S1 had appeared below or it appeared below otherwise.

A second delay (D2) of 0, 400, or 800 ms followed S2. After D2, the 2 visual stimuli reappeared: one 7.8° to the right of the reference point and the other 7.8° to the left, serving as a “go” signal. The monkeys had to choose the stimulus that had been the farthest from the reference point within 6.0 s by pressing the left or right key of the switches. Correct responses were followed by a reward that consisted of 0.1 ml of fluid, whereas incorrect responses were followed by an acoustic signal. A variable intertrial period, usually between 700–1000 ms, separated 2 consecutive trials. The visual stimuli comprised a red square and a blue circle. The duration of the prestimulus periods and delays, the position of the visual stimuli on the screen, their color and shape, and their position (right or left) after the appearance of S2 were pseudorandomly determined.

### Surgery

Recording chambers were implanted over the exposed dura mater of the left frontal lobe, with head restraint devices, using aseptic techniques and isofluorane anesthesia (1% to 3%, to effect). Monkey 1 had 2 18-mm-diameter chambers, and Monkey 2 had a single 27 × 36-mm chamber.

### Data Collection

We recorded eye positions with an infrared oculometer (Arrington Recording) and single cells using quartz-insulated platinum-iridium electrodes (0.5–1.5 MU at 1 kHz), positioned by a 16-electrode drive assembly (Thomas Recording). The electrodes were arranged in a concentric array with 518-μm spacing. Spikes were discriminated online using Multichannel Acquisition Processor (Plexon) and confirmed with Off Line Sorter (Plexon).

### Neural analyses

#### Selection of neurons

From the original dataset^15^, we selected neurons that had a mean activity of at least 1 spike/s in the period between the beginning of the trial and the monkeys’ responses (N = 1443/1765). From this subset, we identified the neurons that encoded the previous outcome (correct vs. incorrect previous trial) in the period between 80 and 400 ms after the central reference point appeared (early prestimulus period). To this end, we first divided the trials by duration of the prestimulus period: 400 (*Pre-S 400* trials) or 800 ms (*Pre-S 800* trials). Then, we performed a one-way ANOVA with mean firing rate activity in the early prestimulus period as the dependent variable and previous outcome as the factor to identify neurons that showed a modulation related to previous outcome for *Pre-S 400* and/or *Pre-S 800* trials. Finally, we selected neurons that experienced a significant modulation in *Pre-S 400* and/or *Pre-S 800* trials and performed a one-way ANOVA, following the same procedure, for 3 additional periods: from 480 to 800 ms after the central reference point appeared (late prestimulus period), from 80 to 400 ms (early S1 period), and from 480 to 800 ms after presentation of S1 (late S1). The late prestimulus and late S1 periods were only used for the *Pre-S 800* trials and *Pre-S 400* trials, respectively.

Neurons that showed a significant outcome modulation in the early prestimulus period of both *Pre-S 400* and *Pre-S 800* trials were further classified as *event-dependent* or *time-dependent* neurons. Neurons that exhibited a significant modulation for the early prestimulus period in *Pre-S 400* trials and in the early and late prestimulus periods of *Pre-S 800* trials but not after presentation of S1 were classified as outcome decline event-dependent neurons (referred to as *event-dependent* neurons). Conversely, neurons that showed a significant outcome modulation for the early prestimulus period in *Pre-S 400* and *Pre-S 800* trials but not in the late prestimulus period of *Pre-S 800* trials and not in the early S1 period in *Pre-S 400* trials were considered outcome decline time-dependent neurons with a 400-ms decline (referred to as *time-dependent 400* neurons). Similarly, neurons that lost the outcome modulation in the late S1 period in the *Pre-S 400* trials and in the early S1 period in the *Pre-S 800* trials were defined as outcome decline time-dependent neurons with an 800-ms decline (referred to as *time-dependent 800* neurons). Thus, *time-dependent 400* neurons ceased to represent the previous outcome 400 ms after the central reference point appeared, whereas *time-dependent 800* ms neurons did so 800 ms after the central point appearance independently of the onset time of S1. In both cases, the dissipation of the previous outcome signal was independent of the appearance of an external event (onset of S1 in our experiment) but dependent on time.

#### Neural response

To compute mean firing rates for the population analyses, we used a temporal window of 50 ms and a sliding window of 5 ms to smooth the curves. All references to time refer to the middle value of the temporal window.

The selectivity index was calculated using the mean spike count (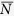) of each neuron in the early prestimulus period, sorted by correct and incorrect previous trials. Then, the condition with the highest neural mean response was identified as the preferred condition (p). Accordingly, the nonpreferred condition (np) was the alternative one:


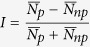


## Additional Information

**How to cite this article**: Marcos, E. *et al.* Event- and time-dependent decline of outcome information in the primate prefrontal cortex. *Sci. Rep.* **6**, 25622; doi: 10.1038/srep25622 (2016).
